# Accuracy of Two Circulating Antigen Tests for the Diagnosis and Surveillance of *Schistosoma mansoni* Infection in Low-Endemicity Settings of Côte d’Ivoire

**DOI:** 10.4269/ajtmh.21-0031

**Published:** 2021-07-19

**Authors:** Rufin K. Assaré, Mathieu I. Tra-Bi, Jean T. Coulibaly, Paul L. A. M. Corstjens, Mamadou Ouattara, Eveline Hürlimann, Govert J. van Dam, Jürg Utzinger, Eliézer K. N’Goran

**Affiliations:** 1 Unité de Formation et de Recherche Biosciences, Université Félix Houphouët-Boigny, Abidjan, Côte d’Ivoire;; 2 Centre Suisse de Recherches Scientifiques en Côte d’Ivoire, Abidjan, Côte d’Ivoire;; 3 Swiss Tropical and Public Health Institute, Basel, Switzerland;; 4 University of Basel, Basel, Switzerland;; 5 Department of Cell and Chemical Biology, Leiden University Medical Center, Leiden, The Netherlands;; 6 Department of Parasitology, Leiden University Medical Center, Leiden, The Netherlands

## Abstract

In low-endemicity settings, current tools for the diagnosis and surveillance of schistosomiasis are often inaccurate in detecting true infection. We assessed the accuracy of an up-converting phosphor lateral flow circulating anodic antigen (UCP-LF CAA) test and a point-of-care circulating cathodic antigen (POC-CCA) urine cassette test for the diagnosis of *Schistosoma mansoni*. Our study was conducted in eight schools of western Côte d’Ivoire. Fifty children, aged 9–12 years, were enrolled per school. From each child, a single urine specimen and two stool specimens were collected over consecutive days for diagnostic work-up. Urine samples were subjected to UCP-LF CAA and POC-CCA tests. From each stool sample, triplicate Kato-Katz thick smears were examined. Overall, 378 children had complete data records. The prevalence of *S. mansoni*, as assessed by six Kato-Katz thick smears, was 4.0%. The UCP-LF CAA and POC-CCA tests revealed *S. mansoni* prevalence of 25.4% and 30.7%, respectively, when considering trace results as positive, and prevalence of 23.3% and 10.9% when considering trace results as negative. In the latter case, based on a composite “gold” standard, the sensitivity of UCP-LF CAA (80.7%) was considerably higher than that of POC-CCA (37.6%) and six Kato-Katz thick smears (13.8%). The negative predictive value of UCP-LF CAA, POC-CCA, and six Kato-Katz thick smears was 92.8%, 79.8%, and 74.1%, respectively. Our results confirm that UCP-LF CAA is more accurate than Kato-Katz and POC-CCA for the diagnosis of *S. mansoni* in low-endemicity settings.

## INTRODUCTION

Schistosomiasis remains a public health problem in many parts of sub-Saharan Africa.^1^^,^^2^ The global burden of schistosomiasis is estimated at 1.4 million disability-adjusted life years (DALYs).^3^ The life cycle of *Schistosoma* requires a human as definitive host, specific freshwater snails as intermediate host, and human-water contacts. Adult *Schistosoma* worms live in the gastrointestinal or urinary tract, where they produce eggs, which pass through stool or urine. Schistosomiasis can lead to anemia, malnutrition, and stunted growth in children, and bladder cancer in adults.^4^

There are several approaches for schistosomiasis control, including snail control; water, sanitation, and hygiene; information, education, and communication; and, most importantly, preventive chemotherapy.^1^ The latter strategy consists of mass drug administration (MDA) with praziquantel, targeting at-risk groups (e.g., school-age children) without prior diagnosis. In 2016, 68.5 million school-age children from 30 African countries received praziquantel.^5^ The implementation of preventive chemotherapy significantly reduced the prevalence of schistosomiasis in many parts of Africa.^6^^,^^7^ In settings where the endemicity of schistosomiasis has been lowered due to preventive chemotherapy, efforts should be made to further reduce the prevalence of heavy-intensity infections to levels < 1% (as determined by egg counts), to eliminate schistosomiasis as a public health problem.^8^ Rigorous monitoring and surveillance in such settings is required so that pockets of transmission can be rapidly identified and tailored public health responses implemented.

Accurate diagnosis is an important prerequisite for monitoring and surveillance of schistosomiasis. With regard to *Schistosoma mansoni*, the Kato-Katz technique is the most widely used approach in epidemiologic surveys assessing the impact of preventive chemotherapy. However, in low-endemicity settings, the Kato-Katz technique lacks sensitivity. Examination of multiple Kato-Katz thick smears, ideally obtained from consecutive stool samples, enhances the sensitivity of the Kato-Katz technique.^9^^,^^10^ However, collection of multiple stool samples is time-consuming and costly and reduces compliance in parasitologic survey. Serological methods, such as ELISA and indirect hemagglutination test, and molecular techniques, such as polymerase chain reaction (PCR) and loop-mediated isothermal amplification are highly sensitive.^11^^–^^13^ Yet, in most schistosomiasis-endemic countries, these diagnostic tests are not available. Hence, there is a need for diagnostic tools that have high sensitivity and specificity and can be available at the point-of-care (POC), particularly in settings where the prevalence and intensity of infection are low.

Prior research has shown that the POC circulating cathodic antigen (CCA) urine cassette test and the up-converting phosphor lateral flow circulating anodic antigen (UCP-LF CAA) assay are more sensitive than the standard Kato-Katz technique for *S. mansoni* diagnosis.^14^^–^^18^ UCP-LF CAA assay has a particularly high sensitivity and specificity, and hence, might be especially well suited for detection of low infection intensities.^19^ Although CAA can be detected both in urine and serum, a urine UCP-LF CAA test can be more sensitive than a serum UCP-LF CAA test due to the adaptability to use large sample volume.^20^^–^^22^ Collection of urine rather than blood usually results in higher compliance.

We report on a study conducted in the western part of Côte d’Ivoire. We purposefully enrolled eight schools where the prevalence of *S. mansoni* based on the Kato-Katz technique, after several rounds of MDA with praziquantel, was very low.^15^ Our objective was to determine the accuracy of UCP-LF CAA, POC-CCA, and multiple Kato-Katz thick smears for the diagnosis of *S. mansoni*.

## METHODS

### Ethics statements.

The study was approved by the Comité National d’Ethique et de la Recherche of the Ministry of Health in Côte d’Ivoire (reference no. 046/MSHP/CNER-kp; date of assignment: May 30, 2016). Before the parasitologic survey, health and education authorities and village leaders received information about the objectives and procedures of the study. Potential risks and benefits were explained to the communities in lay terms. Parents or legal guardians signed a written informed consent, and children provided oral assent. Participation was voluntary, and children could withdraw at any time without further obligation. At the end of the study, all school-age children in the study villages were offered praziquantel free of charge, according to guidelines put forward by the World Health Organization (WHO).^23^

### Study area and population surveyed.

Details of the study area and population surveyed have been described elsewhere.^15^ In brief, we designed a cross-sectional study in 14 schools in three regions in the western part of Côte d’Ivoire, extending from 06°32'42.0'' to 07°36'54.8'' N latitude and from 06°44'09.8'' to 07°33'48.9'' W longitude. The *S. mansoni* prevalence among 9- to 12-year-old children ranged from nil to 22%, as determined by the Kato-Katz technique, and the corresponding values varied from nil to 33%, as assessed by POC-CCA urine cassette test if trace results were considered positive. Eight schools with a low *S. mansoni* prevalence (< 10% according to Kato-Katz) were selected to assess the accuracy of POC-CCA and UCP-LF CAA compared with multiple Kato-Katz thick smears.

### Urine and stool collection.

In each of the eight schools, 50 children aged 9–12 years who had a written informed consent form signed by parents or guardians, were enrolled. The first day of the survey, children received two prelabeled plastic containers and were invited to return the containers filled with a fresh morning stool and a urine sample. Upon collection, children received another prelabeled plastic container and were asked to provide a second stool sample the next day. Stool and urine samples were transferred to a nearby laboratory at the Hôpital Général de Douékoué or the Centre Hospitalier Regional de Man.

### Stool and urine examination.

In the laboratory, stool samples were subjected to triplicate 41.7 mg Kato-Katz thick smears.^24^ Each child provided two stool samples, and in total, six Kato-Katz thick smears per child were examined; the average of the numbers of eggs was used in the analysis. After a clearing time of 30 min, the Kato-Katz thick smears were examined under a microscope by one of four experienced laboratory technicians. Eggs of *S. mansoni*, as well as *Ascaris lumbricoides*, *Enterobius vermicularis*, hookworm, *Hymenolepis* spp., *Taenia* spp., and *Trichuris trichiura*, were counted and recorded for each species separately. Here, only the results pertaining to *S. mansoni* are presented. The cumulative number of *S. mansoni* eggs on the six Kato-Katz thick smears (total volume approximately 250 mg of stool) was multiplied by a factor of 4 to obtain an estimate of infection intensity, proxied by the number of *S. mansoni* eggs per 1 g of stool (EPG). Infection intensity of *S. mansoni* was stratified into three categories: 1) light (1–99 EPG), 2) moderate (100–399 EPG), and 3) heavy (≥ 400 EPG) according to guidelines put forward by WHO.^25^

Urine samples were tested using the POC-CCA assay (Rapid Medical Diagnostics; Pretoria, South Africa; batch number: 50182).^26^ In brief, one drop of urine was added to the well of the cassette test. Next, one drop of test buffer was added, according to the manufacturer’s protocol (note that the currently offered POC-CCA test format is adapted for use without buffer). The cassette test was read after 20 min by an experienced laboratory technician and results were categorized as negative, trace, or positive.

*S. haematobium* eggs were identified and quantified using urine filtration method. In addition, an aliquot of 5 mL of urine was frozen. The aliquots were transferred to a laboratory at the Centre Suisse de Recherches Scientifiques en Côte d’Ivoire (Abidjan, Côte d’Ivoire) and stored at –20°C. Frozen urine samples were shipped to the Department of Parasitology, Leiden University Medical Center (LUMC; Leiden, The Netherlands) and kept at –20°C pending further analysis.

At LUMC, urine samples were subjected to UCP-LF CAA. The UCP-LF CAA test was performed using 2 mL of urine (UCAA2000, wet assay format), which allows detecting 0.1 pg/mL CAA.^27^^,^^28^ The results of the UCAA2000 assay were stratified according to two approaches. For the first approach (fine-grained classification), the results were stratified into six categories as follow: 1) urine sample is considered high positive at CAA levels of > 100 pg/mL, 2) positive at 10 to 100 pg/mL, 3) low positive at 1 to 10 pg/mL, 4) very low positive at 0.1 to 1 pg/mL, 5) indecisive at 0.05 to 0.1 pg/mL, and 6) negative at CAA levels of < 0.05 pg/mL. In the second approach (coarser classification), the UCAA2000 results were stratified into three categories: 1) urine sample considered positive at CAA levels of > 0.1 pg/mL, 2) indecisive at 0.05 to 0.1 pg/mL, and 3) as negative at < 0.05 pg/mL. Of note, 50% of indecisive results may be true positives when tested with larger sample volume.^21^ A sample returning a CAA level in the indecisive range in this study may be indicated as a trace result for conformity with the POC-CCA terminology.

### Statistical analysis.

Data were entered into Microsoft Excel 2010 (Microsoft Corporation, Redmond, WA) and analyzed with STATA version 15.1 (Stata Corporation, College Station, TX). In the absence of a diagnostic “gold” standard, we defined a composite “gold” standard, as described elsewhere.^29^ In short, a child was considered positive when at least one *S. mansoni* egg was found on any of the six Kato-Katz thick smears, and/or a positive POC-CCA, and/or a positive UCP-LF CAA test result was obtained. POC-CCA trace and UCP-LF CAA indecisive results were considered as negative. On the basis of this approach, sensitivity and negative predictive value (NPV) of the diagnostic tools were determined. The nonparametric Spearman’s rank correlation test was applied to measure the correlation between CAA pg/mL levels and the fecal egg count (expressed in EPG) or POC-CCA scores (negative, trace, and positive).

The strength of agreement between the diagnostic tests was assessed by Kappa statistics (κ).$\text{ In brief }\kappa =\frac{po-pe\;}{1-pe}$, where po is the observed proportion of agreement and pe is the proportion of agreement expected by chance. κ = 0 indicates no agreement; κ = 0.0 to 0.2 indicates poor agreement; κ = 0.21 to 0.4 indicates fair agreement; κ = 0.41 to 0.6 indicates moderate agreement; κ = 0.61 to 0.8 indicates substantial agreement; and κ = 0.81 to 1.0 indicates almost perfect agreement.^30^ The same degrees of agreement were applied for the Spearman rank correlation.

## RESULTS

### Population characteristics.

**Figure 1.**
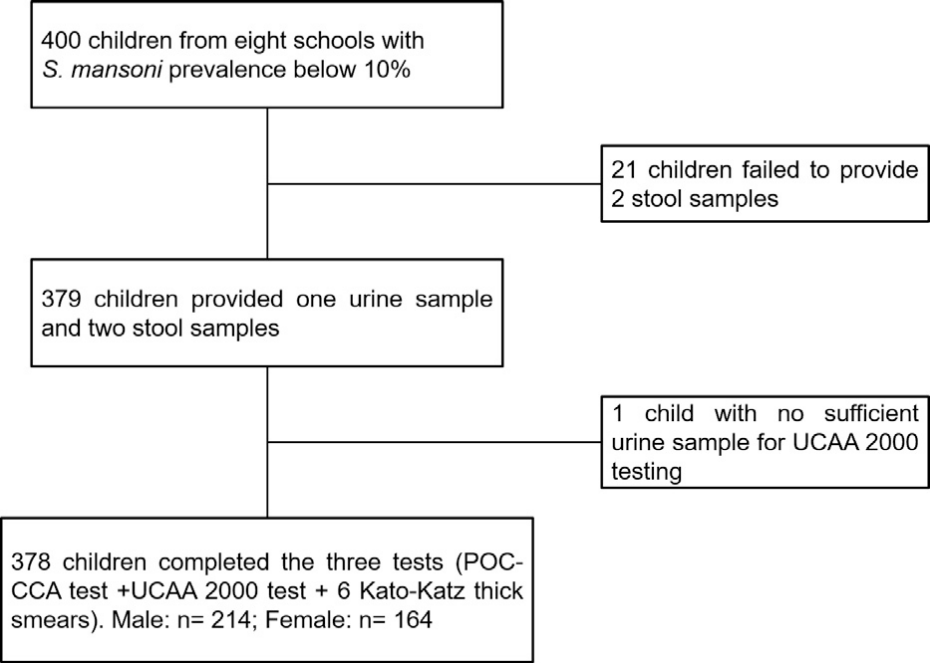
Flow chart detailing study participation.

A total of 400 children, aged 9–12 years from eight schools, had informed consent signed by their parents or guardians, and hence, they were invited to participate. Twenty-one children failed to provide two stool samples over consecutive days, and one child had an insufficient amount of urine for the UCP-LF CAA test. Taken together, 378 children had complete data records (i.e., two stool samples, each subjected to triplicate Kato-Katz thick smears, and one urine sample subjected to both POC-CCA and UCP-LF CAA) (Figure 1). The mean age of the 378 children was 10.4 years with considerably more boys than girls (214 versus 164).

### *S. mansoni* prevalence according to different diagnostic techniques.

Table 1 summarizes the prevalence of *S. mansoni,* stratified by school (village) and the diagnostic test used. Stool examination with six Kato-Katz thick smears revealed a *S. mansoni* prevalence of 4.0% (15/378) with a range at the unit of the school from nil (Gregbeu) to 6.3% (Semien). The intensity of *S. mansoni* infection, expressed as arithmetic mean among *S. mansoni* egg-positive children, was 49.1 EPG (95% confidence interval [CI]: 20.0–78.1 EPG). The lowest mean intensity of infection amongst egg-positive children was 4.0 EPG (Siambly), and the highest value was 88.0 EPG (Mahinahi). Two children with the highest infection level would fall in the moderate infection intensity (100 and 192 EPG) according to WHO definition.

**Table 1:** Prevalences of *Schistosoma mansoni* among children aged 9–12 years in eight villages of western Côte d’Ivoire, stratified by diagnostic approaches

		Sextuplicate Kato-Katz		UCP-LF CAA Indecisive positive		UCP-LF CAA Indecisive negative		POC-CCA Trace positive		POC-CCA Trace negative	
School	No. of children examined	No. positive	%	No. positive	%	No. positive	%	No. positive	%	No. positive	%
Gregbeu	47	0	0.0	7	14.9	6	12.8	11	23.4	11	23.4
Klangbolably	50	3	6.0	9	18.0	9	18.0	16	32.0	4	8.0
Mahinahi	36	1	2.8	10	27.8	9	25.0	15	41.6	7	19.4
Mona	47	2	4.3	11	23.4	11	23.4	17	36.2	6	12.8
Semien	48	3	6.3	11	22.9	9	18.8	10	20.8	4	8.3
Siambly	50	1	2.0	6	12.0	4	8.0	12	24.0	1	2.0
Zouatta II	50	3	6.0	31	62.0	30	60.0	22	44.0	5	10.0
Zê	50	2	4.0	11	22.0	10	20.0	13	26.0	3	6.0
Total	378	15	4.0	96	25.4	88	23.3	116	30.7	41	10.9

CAA = circulating anodic antigen; CCA = circulating cathodic antigen; POC = point-of-care; UCP-LF = up-converting phosphor lateral flow.

On the basis of UCP-LF CAA test, there were 74.6%, 2.1%, 10.6%, 5.3%, 2.7%, and 4.8% of negative, indecisive, very low positive, low positive, positive, and high positive results, respectively. Considering a coarser classification, there were 74.6%, 2.1%, and 23.3% negative, indecisive, and positive results. When considering indecisive (trace) results as positive, the prevalence of *S. mansoni* was 25.4% (96/378), ranging between 12.0% (Siambly) and 62.0% (Zouatta II). Considering indecisive results as negative, the *S. mansoni* prevalence was slightly lower (23.3%), ranging from 8.0% (Siambly) to 60.0% (Zouatta II). The UCP-LF CAA test (UCAA2000 format) revealed a more than 5-fold higher *S. mansoni* prevalence compared with six Kato-Katz thick smears.

Considering trace results as positive, there were 116 (30.7%) children with a positive POC-CCA urine cassette test result. At the unit of the school, the prevalence ranged from 20.8% (Semien) to 44.0% (Zouatta II). The overall prevalence decreased to 10.9% when POC-CCA trace results were counted as negative with the lowest prevalence of 2.0% observed in Siambly and the highest prevalence of 23.4% estimated for Gregbeu.

On the basis of a single urine filtration, *S. haematobium* eggs were observed in only two children (0.5% overall), including one child in Zê (2%) and one child in Zouatta II (2%). In Zê, the urine filtration–positive child was also positive by Kato-Katz, POC-CCA, and UCP-LF CAA. In Zouatta II, the *S. haematobium* egg-positive child was also positive by Kato-Katz and UCP-LF CAA, but negative by POC-CCA.

### Diagnostic accuracy.

Table 2 shows the accuracy of the individual diagnostic assays when considering the composite test as diagnostic “gold” standard. We determined a sensitivity of 80.7%, 37.6%, and 13.8% for UCP-LF CAA, POC-CCA, and six Kato-Katz thick smears, respectively. The combination of UCP-LF CAA and POC-CCA together had the highest sensitivity (97.2%). The NPV was 92.8%, 79.8%, and 74.1% for UCP-LF CAA, POC-CCA, and six Kato-Katz thick smears, respectively. The combination of UCP-LF CAA and POC-CCA revealed higher NPV (98.9%) than UCP-LF CAA and Kato-Katz (93.7%) or POC-CCA and Kato-Katz (81.0%).

**Table 2:** Sensitivity and NPV of different tools for diagnosis of *Schistosoma mansoni*

Diagnostic approach*	Sensitivity	NPV
Kato-Katz (6×)	13.8	74.1
95% CI	7.9–21.7	69.3–78.5
UCP-LF CAA†	80.7	92.8
95% CI	72.1–87.7	89.1–95.5
POC-CCA‡	37.6	79.8
95% CI	28.5–47.4	75.1–84.0
UCP-LF CAA + Kato-Katz (6×)	83.5	93.7
95% CI	75.2–89.9	90.3–96.2
UCP-LF CAA + Kato-Katz (6×)	42.2	81.0
95% CI	32.8–52.0	76.4–85.1
UCAA2000 + POC-CCA	97.2	98.9
95% CI	92.2–99.4	96.8–99.8

CAA = circulating anodic antigen; CCA = circulating cathodic antigen; CI = confidence interval; NPV = negative predictive value; POC = point-of-care; UCP-LF = up-converting phosphor lateral flow.

* Gold standard: Participant considered positive if he was found *S. mansoni* egg positive and/or found UCP-LF CAA test positive and/or POC-CCA test positive.

† Considered UCP-LF CAA indecisive as negative results.

‡ Considered POC-CCA trace negative results.

### Correlation and agreement between diagnostic assays.

**Figure 2.**
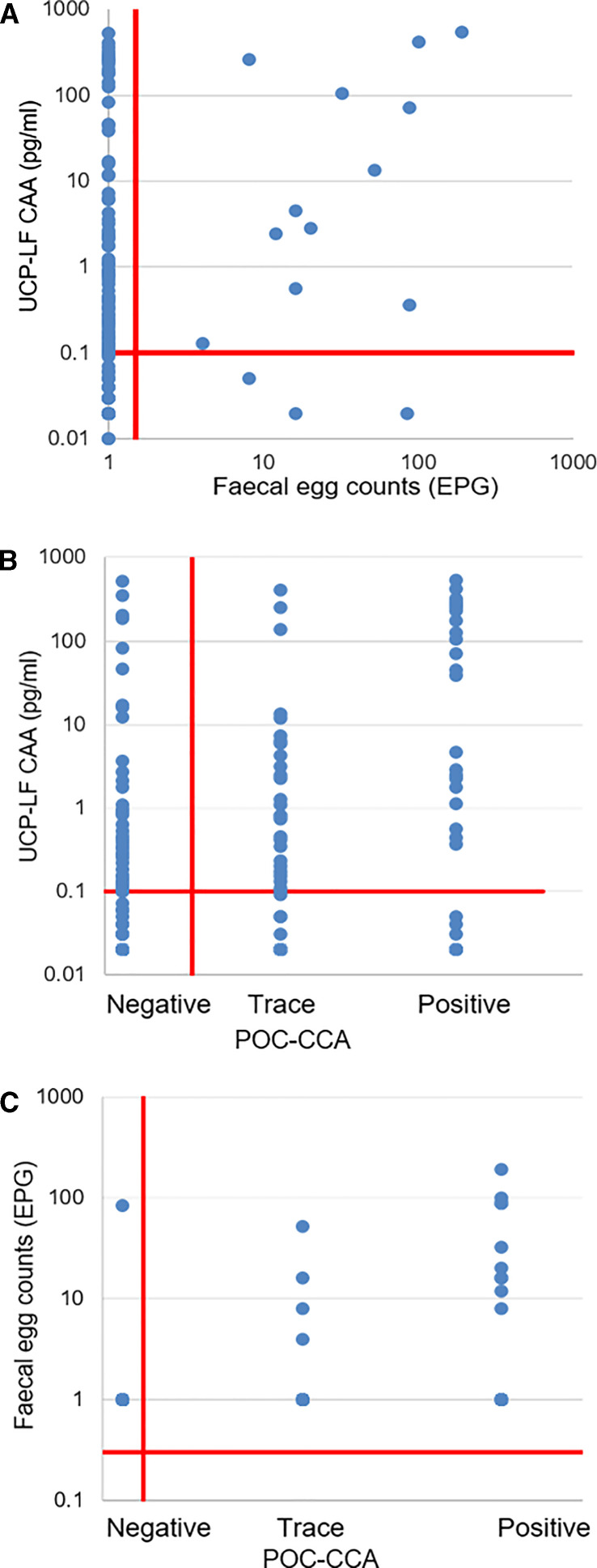
Correlation of *Schistosoma mansoni* urine circulating anodic antigen (CAA) levels and fecal egg counts (EPG) or point-of-care (POC)-CCA intensity scores. (**A**) Correlation of CAA levels (pg/mL) determined by UCAA2000 and fecal egg counts (mean EPG of at least three slides). (**B**) Correlation of CAA levels (pg/mL) and POC-CCA intensity scores. (**C**) Correlation of fecal egg counts and POC-CCA.

There was a fair, yet significant positive relationship between urine CAA levels (pg/mL) and fecal *S. mansoni* egg counts (Spearman’s rho = 0.29; *P* < 0.001), between CAA concentrations and POC-CCA score (Spearman’s rho = 0.35; *P* < 0.001), and between *S. mansoni* egg counts and POC-CCA score (Spearman’s rho = 0.32; *P* < 0.001) (Figure 2).

Table 3 summarizes the number of positive and negative results for diagnostic assay combinations and the agreement between diagnostic techniques. Considering UCP-LF CAA indecisive and POC-CCA trace results as negative, a fair agreement between these two assays was found (κ = 0.24, *P* < 0.001). The agreement between POC-CCA and six Kato-Katz thick smears was also fair (κ = 0.32, *P* < 0.001). The agreement between UCP-LF CAA test and six Kato-Katz thick smears was poor (κ = 0.18, *P* < 0.001).

**Table 3:** Agreement between the different diagnostic methods

UCP-LF CAA				
Kato-Katz (6×)	Positive	Negative	Total	Kappa
Positive	12	3	15	
Negative	76	287	363	
Total	88	290	378	0.18
POC-CCA				
Kato-Katz (6×)	Positive	Negative	Total	Kappa
Positive	10	5	15	
Negative	31	332	363	
Total	41	337	378	0.32
UCP-LF CAA				
POC-CCA	Positive	Negative	Total	Kappa
Positive	23	65	88	
Negative	18	272	290	
Total	41	337	378	0.24

CCA = circulating cathodic antigen; POC = point-of-care; UCP-LF CAA = up-converting phosphor lateral flow circulating anodic antigen. Trace POC-CCA and indecisive UCP-LF CAA considered negative.

## DISCUSSION

Preventive chemotherapy with praziquantel is the current mainstay of schistosomiasis control. This strategy controls morbidity and might impact on the force of transmission, thus also playing a role in disease elimination in specific settings.^6^^,^^7^^,^^31^ In epidemiologic surveys, the diagnosis of schistosomiasis is usually based on stool and urine microscopy, which is inappropriate in low endemicity settings. Indeed, copro-microscopic approaches fail to detect light-intensity infections that are particularly common after several rounds of MDA with praziquantel.^13^ Hence, there is a need for new diagnostic tools with high specificity and sensitivity in low-endemicity settings.

The current study assessed the accuracy of POC-CCA and UCP-LF CAA for the diagnosis of *S. mansoni* among 9- to 12-year-old children in eight schools in the western part of Côte d’Ivoire that remained endemic for *S. mansoni* despite 5 years of preventive chemotherapy.^15^^,^^32^ The two urine-based assays detect pathogen-derived antigens. Similar to the detection of *Schistosoma* eggs, circulating antigens indicate an ongoing active infection, with the concentration of antigen directly linked to the *Schistosoma* worm burden. The overall *S. mansoni* prevalence was lower than 5% by six Kato-Katz thick smears derived from two consecutive stool samples. Urine samples assayed by the UCP-LF CAA test demonstrated a prevalence of 25.4%, or 23.3% when indecisive results were considered negative. On the basis of the POC-CCA urine cassette test results, the *S. mansoni* prevalence was 30.7%, or 10.9% when trace results were considered negative. The sensitivity of the UCP-LF CAA test was 80.7%, whereas the corresponding sensitivity of POC-CCA and Kato-Katz assays were much lower; 37.6% and 13.8%, respectively. In two children from different schools, *S. haematobium* eggs were detected with no indication of cross-reactivity with circulating antigens for *S. mansoni*.

We found that the two urine circulating antigen tests (UCP-LF CAA and POC-CCA) revealed several-fold higher *S. mansoni* prevalence than stool microscopy with multiple Kato-Katz thick smears. Indeed, when indecisive results were considered positive, the UCP-LF CAA test revealed a 6.4-fold higher prevalence of *S. mansoni* compared with Kato-Katz. Considering indecisive results as negative, there was still a 5.8-fold higher prevalence estimate. Interestingly, in the Gregbeu school where no *S. mansoni* positive case was observed by Kato-Katz, the prevalence measured by UCP-LF CAA assay was still above 10%, regardless of whether indecisive results were included. The POC-CCA test revealed a prevalence of 23.4%. Our observations confirm that circulating antigen tests have a much higher sensitivity for *S. mansoni* diagnosis than stool microscopy, particularly in low-endemicity settings. Our findings corroborate results from rural parts of northeastern Brazil and five ecological zones in Burundi.^17^^,^^33^ Moreover, our observations are in line with results of an analysis of banked urine samples from Cambodia and the Philippines for detection of *S. mekongi* and *S. japonicum,* respectively.^34^ In addition, a recent study comparing novel and standard diagnostic techniques for *S. mekongi* infection in Lao People’s Democratic Republic and Cambodia reported that the UCP-LF CAA (UCAA2000 format) detected more *S. mekongi* cases than POC-CCA and multiple Kato-Katz thick smears.^19^

Our results also confirmed that POC-CCA urine cassette test is more sensitive than multiple Kato-Katz thick smears, yet the sensitivities were low against our composite “gold” standard (37.6% and 13.8%, respectively).^14^^,^^35^ A recent study conducted in a village in Kafr El Dewar province in Egypt revealed that the sensitivity of POC-CCA was significantly higher than that of a PCR technique for *S. mansoni* diagnosis.^36^ The main strengths of the POC-CCA urine cassette test are the relative low cost, simplicity of use, and the short duration until results are available compared with UCP-LF CAA test, which still requires a basic laboratory setup. The POC-CCA has been recommended for mapping schistosomiasis mansoni at large scale.^37^

Since our study, the format of the POC-CCA has been changed, and some issues with batch-to-batch variation have been reported.^38^ This mainly applies to the very low prevalence areas in Brazil^39^; in low-to-moderate areas, the POC-CCA provides a more accurate estimate of the true prevalence of *S. mansoni* compared with Kato-Katz thick smear readings.^40^ Caution must always be taken with the use of different production batches of rapid diagnostic tests, comparing the performance of consecutive batches using a set of reference samples.

We found that the combination of UCP-LF CAA and POC-CCA showed high sensitivity for *S. mansoni* diagnosis; indeed, the sensitivity was close to 100%. UCP-LF CAA indecisive and POC-CCA trace results are shortcomings when using circulating antigen assays. In case of the UCP-LF CAA test, indecisive samples in principle can be retested using a larger volume to get a definite result. Clearly, when pushing LF–based assays for highest sensitivity, trace results will always be an issue, especially with visual scored tests as the POC-CCA. Reader-assisted tests like the UCP-LF CAA can omit trace (indecisive) results by setting a proper positivity threshold. In this study, data were analyzed considering trace (indecisive) results either as positive or negative.

## CONCLUSION

Accurate diagnostic tools are pivotal for schistosomiasis risk profiling, measuring progress of gaining and sustaining control, and verification of elimination. Our research contributes to further evaluation of the accuracy of UCP-LF CAA for *S. mansoni* diagnosis. We found that UCP-LF CAA test (i.e., UCAA2000 format) is more sensitive than POC-CCA and Kato-Katz thick smears. Hence, the UCAA2000 format is a promising tool for diagnosis, particularly in low-endemicity settings. Compared with the POC-CCA test, the number of indecisive (trace) results are much fewer. However, the CAA-based test remains a laboratory-dependent tool and is not commercially available in low- and middle-income countries, where schistosomiasis is a public health problem and continues to impair the social and economic development. It is recommended to focus further on improving its affordability and field-friendly (POC) applicability.
